# Supplementary material for: MetaRibo-Seq measures translation in microbiomes
Source: Nat Commun. 2020 Jun 29;11:3268. doi: 10.1038/s41467-020-17081-z (PMC7324362; doi:10.1038/s41467-020-17081-z)
Supplement: Supplementary file 10 — Supplementary Data 7 [file 41467_2020_17081_MOESM10_ESM.zip › File2/Confidence_High_Taxonomy/7336_out.krona.html]

Javascript must be enabled to view this page.

members
magnitude
magnitudeUnassigned
count
unassigned
taxon
rank

7336\_out

2

2
2
superkingdom

1239
2
phylum

186801
2
class

186802
order
2

186803
family
2

2
1

SRS1041036\_contig\_number\_contig-100\_18907.18908
genus
841

species
1
166486

SRS016954\_contig\_number\_contig-100\_38134.67272
